# Supplementary material for: Development of a computer-aided design software for the quantitative evaluation of aesthetic damage
Source: PLoS One. 2019 Dec 18;14(12):e0226322. doi: 10.1371/journal.pone.0226322 (PMC6919621; doi:10.1371/journal.pone.0226322)
Supplement: S1 Code site — 1. (ZIP) [file pone.0226322.s001.zip › [EESC_jr][Projeto]Relatorio_07_21/painel_es/index_antigo.php]

Informes AIPE

php
include("header.php");
?


Total Users

2500

*4%*  From last Week

Average Time

123.50

*3%*  From last Week

Total Males

2,500

*34%*  From last Week

Total Females

4,567

*12%*  From last Week

Total Collections

2,315

*34%*  From last Week

Total Connections

7,325

*34%*  From last Week

### Network Activities Graph title sub-title

December 30, 2014 - January 28, 2015

## Top Campaign Performance

Facebook Campaign

Twitter Campaign

Conventional Media

Bill boards

  

## App Versions

- - Settings 1
  - Settings 2

#### App Usage across versions

0.1.5.2

60% Complete

123k

0.1.5.3

60% Complete

53k

0.1.5.4

60% Complete

23k

0.1.5.5

60% Complete

3k

0.1.5.6

60% Complete

1k

## Device Usage

- - Settings 1
  - Settings 2

| Top 5 | Device  Progress |
| --- | --- |
|  | IOS \| 30%; Android \| 10%; Blackberry \| 20%; Symbian \| 15%; Others \| 30% |

## Quick Settings

- - Settings 1
  - Settings 2

- Settings
- Subscription
- Auto Renewal
- Achievements
- Auto Renewal
- Achievements
- Logout

#### Profile Completion

$
3,200
$5,000

## Recent Activities Sessions

- - Settings 1
  - Settings 2

- ## Who Needs Sundance When You’ve Got Crowdfunding?

  13 hours ago by Jane Smith

  Film festivals used to be do-or-die moments for movie makers. They were where you met the producers that could fund your project, and if the buyers liked your flick, they’d pay to Fast-forward and… Read More
- ## Who Needs Sundance When You’ve Got Crowdfunding?

  13 hours ago by Jane Smith

  Film festivals used to be do-or-die moments for movie makers. They were where you met the producers that could fund your project, and if the buyers liked your flick, they’d pay to Fast-forward and… Read More
- ## Who Needs Sundance When You’ve Got Crowdfunding?

  13 hours ago by Jane Smith

  Film festivals used to be do-or-die moments for movie makers. They were where you met the producers that could fund your project, and if the buyers liked your flick, they’d pay to Fast-forward and… Read More
- ## Who Needs Sundance When You’ve Got Crowdfunding?

  13 hours ago by Jane Smith

  Film festivals used to be do-or-die moments for movie makers. They were where you met the producers that could fund your project, and if the buyers liked your flick, they’d pay to Fast-forward and… Read More

## Visitors location geo-presentation

- - Settings 1
  - Settings 2

## 125.7k Views from 60 countries

|  |  |
| --- | --- |
| United States | 33% |
| France | 27% |
| Germany | 16% |
| Spain | 11% |
| Britain | 10% |

## To Do List Sample tasks

- - Settings 1
  - Settings 2

- Schedule meeting with new client
- Create email address for new intern
- Have IT fix the network printer
- Copy backups to offsite location
- Food truck fixie locavors mcsweeney
- Food truck fixie locavors mcsweeney
- Create email address for new intern
- Have IT fix the network printer
- Copy backups to offsite location


## Daily active users Sessions

- - Settings 1
  - Settings 2

**Monday**, 07:30 AM
F
**C**

## Texas *Partly Cloudy Day*

### 23

## Mon

### 25

##### 15 *km/h*

## Tue

### 25

##### 12 *km/h*

## Wed

### 27

##### 14 *km/h*

## Thu

### 28

##### 15 *km/h*

## Fri

### 28

##### 11 *km/h*

## Sat

### 26

##### 10 *km/h*

php
require("footer.php");
?
